# Multiple Bone and Joint Diseases in a Nigerian Sickle Cell Anaemia: a Case Report

**DOI:** 10.4084/MJHID.2012.023

**Published:** 2012-05-07

**Authors:** John A. Olaniyi, Adekunle E. Alagbe, Toluwalase A. Olutoogun, Oluwasogo E. Busari

**Affiliations:** Department of Haematology, University College Hospital, Ibadan, Nigeria

## Abstract

This case highlights the fact that bone involvement is the commonest clinical manifestation of Sickle Cell Disease (SCD) both in the acute settings such as painful vaso-occlusive crisis (VOC) and as a source of chronic, progressive debility such as avascular necrosis (AVN), chronic osteomyelitis and fixed flexion deformity of joints.

Protracted multiple bone involvement i.e. bilateral femoral and left humeral chronic osteomyelitis, Left elbow, Left knee and right humeral septic arthritis together with avascular necrosis of both femoral and right humeral heads, coupled with urinary tract infection (UTI) and decubitus ulcer in a young adult SCD patient is an unusual occurrence. This morbidities resulted into an uninterrupted 29 weeks of hospitalization for the patient who had previously enjoyed fairly good health.

Various micro-organisms were sequentially cultured at various times and sites; these include E coli and Klebsiella in urine and klebsiella spp in the aspirates of the affected knee joint, elbow joint and femoral osteomyelitis. A screen for HIV 1 and 2 were non-reactive.

Multidisciplinary approach was applied to the patient who was finally discharged home on a wheelchair. This case reflects not only the high susceptibility of SCD patients to infection, but also the morbidity and the attendant complications. It also highlights the need to forestall VOC which often predisposes to osteomyelitis. There is a need to have a highly organized, well-equipped and highly subsidized Sickle Cell and rehabilitation center in Nigeria in order to improve the medical care for SCD patients.

## Introduction

Painful vaso-occlusive crises (VOC) and osteomyelitis are the most frequent complications requiring hospital admissions for patients with sickle cell disease (SCD).^1^,^2^ Malaria, bacterial and other forms of infections are associated with crises, exacerbation of morbidity and poor survival among patients with SCD.^3^,^4^

The unique case of this 20 year old patient with HbSS SCD who walked in with severe bone pain crisis but was finally discharged home on a wheelchair after 29 weeks of turbulent hospital admission clearly demonstrated the impact of widespread bone marrow micro vascular occlusion and increased susceptibility to infections, especially in patients with more severe haplotypes like Benin and Senegal to which Nigeria belongs.^5^,^6^,^7^

This case is presented not only to showcase devastating bone complications of SCD but to further highlight the pathophysiologic mechanisms involved viz: the role of VOC, abdominal crises and haematogenous spread of micro-organisms in causing osteomyelitis and septic arthritis and their cumulative impact of escalating morbidity and mortality in SCD patients.

## Case Presentation

She is a 20 y. o., single, known HbS, a polytechnic student who complied well with clinic attendance and routine medications. She was admitted with severe infarctive crisis and anemic heart failure, keeping in view background sepsis as the precipitating factor.

She presented with a week history of severe bone pains involving lower limbs, lower back and both upper limbs, generalized abdominal pain, non-projectile postprandial vomiting (4 episodes), high grade intermittent fever, passage of dark urine and deep yellowness of the eye. There was history of recurrent hip pain since age 13yrs and x-ray then confirmed avascular necrosis (AVN) of the left femoral head which was being managed conservatively. Frequency of bone pains crisis had been 1–2 per year and she was transfused only once in the past.

Physical findings revealed an acutely ill looking young lady in painful distress, severely pale, afebrile (Temp. 36.6°C), severely icteric, mildly dehydrated, not cyanosed, with no pedal edema. She was dyspnoeic (RR-44pm) with vesicular breath sounds. She was tachycardic (PR-100bpm) with gallop rhythm (S1,S2,S3), and BP-120/60mmHg. Abdomen was soft with tender hepatomegaly of 6 cm below RCM MCL. Central Nervous system was grossly intact while musculo-skeletal system showed shortening of the right lower limb and diffuse mild tenderness of both lower limbs from the waist to the toes. She was then managed as SCD with infarctive crisis and anemic heart failure, keeping in view background sepsis.

The immediate FBC showed PCV of 13%, WBC of 50,000/cmm, Platelet count of 180,000/cmm. Blood smear showed numerous target cells, admixture of macrocytes and microcytes, 6% irreversible sickle cells, moderate hypochromia and circulating megaloblasts (40 nucleated red cells / 100 white cells, leucocytosis (Corrected WBC was 50,000/cmm), granulocytes showed left shift with toxic granulations but there were also hypersegmented neutrophils. Platelets were adequate. These features were in keeping with combined (iron and folate) deficiency anemia and sepsis in a SCD patient.

On account of PCV of 13% she was transfused with 3 units of O Rh D positive compatible packed red cells, a unit per day, over three days. Broad spectrum antibiotics were commenced along with analgesia and intranasal oxygen together with generous intravenous hydration.

Biochemical findings essentially showed acidosis (Bicarbonate-15mmol/l) and hyperazotemia (Urea-103mg/dl). The Nephrologist reviewed along with renal USS features consistent with bilateral grade II renal parenchyma disease and concluded on acute kidney injury which was managed conservatively.

Her condition was critical over the first five days of admission as she subsequently became febrile, continued to pass dark brown urine, developed severe pains involving the ribs and also developed severe abdominal pain and distention with associated vomiting (? abdominal crisis). Therefore, patient was placed on “nil per oral”, and fluid was supplemented with 40 mls of 50% d/w per each liter of fluid, I.V. Augmentin was changed to I.V. Ceftriaxone 1g 12hrly. Abdominal USS only confirmed hepatomegaly of 19 cm span. The patient was non-reactive to HIV 1&2, hepatitis B and C screening, blood film for malaria parasite was negative.

She then had another top-up transfusion which raised her PCV to 23%. Repeat electrolytes showed mainly mild hypokalaemia (K-2.4 mmol/l) with normal bicarbonate (24 mmol/l) and urea (63mg/dl). The hypokalaemia was corrected using half-strenght Darrows infusion. Patient passed semi-solid brown stool for the first time on day 7 of admission.

**Figure 1 and 2.**
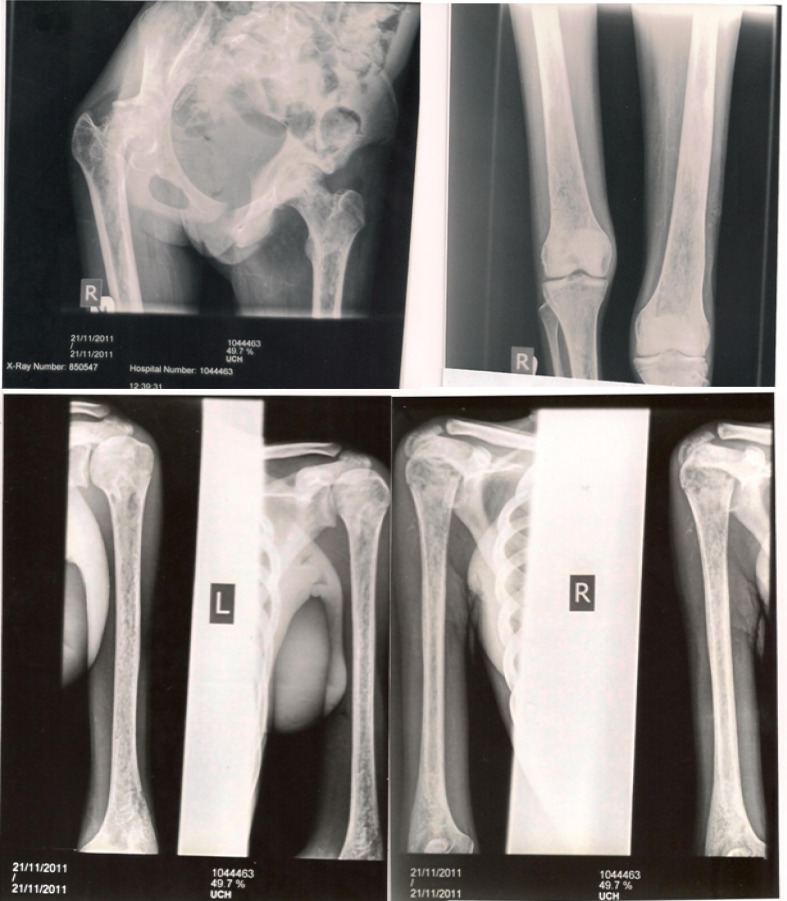
**Radiologists report** *Right shoulder:* Ill-defined cystic changes, cortical thickening laterally with the shaft of humerous and soft tissue swelling in keeping with osteomyelitis of humeral shaft. *Pelvis:* Pelvic asymmetry, flattening of the lateral half of the right femoral head with some lucencies seen. Mixed sclerotic and cystic changes are noticed in the neck. *Both femour:* There is sclerosis of mid shaft of left femur with some background cystic changes and edosteal reactions. The right middle half showed thickening of cortex with soft tissue swellin of thigh. These are in keeping with bilateral chronic osteomyelitis(Rt>>Lt)

By the second week, watery stooling started, pain was less, fever persisted and stool for MCS was negative for bacteria, ova and parasite. A markedly tender, warm distal 3^rd^ of the right femur was observed and x-ray film was suggestive of acute osteomyelitis. In addition, X-ray film of the pelvis showed evidence of AVN of both femoral heads (left worse than right) and chronic osteomyelitis of the femoral bones (**Refer figure 1 and 2**). At this point antibiotics were converted to I.V. Floxapen and Ciprofloxacilin.

By the third week, fever persisted but signs of hyperhaemolysis abated. She however had residual pain in the right shoulder, left elbow, lower back, and both femurs with inability to sit or walk. At this point antimalaria-(I.M. paluther) was administered. Chest X-Ray film showed left ventricular hypertrophy, and fluffiness of lung fields with increased bronchovascular markings.

In view of persistent fever and multiple bone abnormalities, the microbiologist and orthopaedic surgeon were consulted. Blood culture yielded E.coli; and also Urine culture yielded klebsiella Spp. and antibiotics to which the respective organisms were sensitive were commenced.

The sequence of events and interventions from the 5^th^ week to the 29^th^ week of discharge is as summarized. Weeks 5–16 of hospital admission was characterized by sequential eruption of foci of infections like left elbow and knee septic arthritis (aspirate grew klebsiella spp), bed sores, urinary tract infection (UTI) [ urine culture grew klebsiella spp and staph aureus]. As determined by isolated organism, antibiotic sensitivity and in line with Microbiologists advice, appropriate antibiotics were prescribed ranging from Cefuroxime, Amikacin, Augmentin, Metronidazole. All through this period high grade fever persisted (Temperature 39° C) and hence at week 13 Imipenem was given at 500mg in 100 mls of normal saline (run over 30 minutes) every 6 hours for 5 days and by week 16 the patient became afebrile but pus continue to drain from septic sites- left thigh osteomyelitic site and left knee septic arthritis site.

Weeks 17–29 of admission was characterized by multiple joint stiffness and tenderness,muscular atrophy and rigidity for which the services of the Physiotherapists and palliative team were required.

Many FBC carried out at this period consistently showed leukocytosis (17,000 – 23,000/cmm) and thrombocytosis (546,000 –637,000/cmm). In view of these, hydroxyurea was started at 500mg daily at week 20 of admission and continued after discharge. Apart from minimal blood-serum secretion from the right femur and UTI which recurred (urine culture yielding Proteus spp) and which was treated using Nitrofurantoin, patient remained fairly stable until she was discharged at week 29 of hospital admission on a wheelchair.

## Discussion

Musculoskeletal manifestations is the commonest clinical manifestation of SCD both in the acute setting in form of painful VOC and as a source of chronic, progressive disability such as AVN, chronic osteomyelitis and septic arthritis.

The clinical course of a SCD patient is typically characterized by chronic haemolytic anemia, variable periods of steady states interrupted by painful vaso-occlusive crisis, which can be triggered by psychological, physical and infective factors.^5^,^6^ It is equally characterized by high susceptibility to infections like osteomyelitis, a major skeletal complication in SCD patients.^8^,^9^ However, distinction between acute bone infarction and acute osteomyelitis in patients with SCD remain one of the most difficult diagnostic dilemmas in the management of SCD since fever, localized bone pain, and localized erythema, swelling and tenderness are characteristic of both conditions. So also, both typically have an elevated ESR. However, the positive blood culture favors the diagnosis of acute osteomyelitis. In this index patient, although background sepsis was suspected ab initio, it took 2 weeks before the first site of osteomyelitis became obvious and subsequent blood and aspirate culture grew E. coli and Klebsiella respectively. The three main causes of bone and joint involvement in SCD are: a) bone marrow hyperplasia causing distortion and growth disturbance, b) vaso-occlusive events that lead to infarction of metaphyseal and diaphyseal bone and to osteonecrosis of juxta-articular bones and c) haematogenous bacterial infection that results in osteomyelitis and septic arthritis.^10^ While (b) may explain the femoral and humeral head necrosis in this patient, haematogenous bacteria infection (c) appears to take prominence in this index patient in that severe abdominal crisis which the patient presented with among others could allow seepage of normal gut flora into the blood stream. Haematogenous spread is possible because SCD patients have sluggish circulation of blood in bones and also have compromised immune status attributable to many inherent factors such as defective microbial opsonization, tissue infarction and splenic hypofunction.^11^,^12^

Other potential mechanisms through which infections may lead to VOC involve several pathological changes like, pyrexia, acute phase reaction, hypercoagulability, neutrophilia, eosinophilia, thrombocytosis,, red cell cytopathic and membrane changes as in malaria, diarrhoea and vomiting, which may act singly or in concert to cause red cell sickling. These changes do induce sickling directly or indirectly through their adverse effects on haemoglobin oxygenation and polymerization, hydration, blood viscosity, red cell metabolism, pro-coagulant activation, intercellular adherence and aggregation, culminating in VOC.^11^,^12^

In this patient, apart from local tenderness, warmth and swelling of bones and joints, there were also persistent high grade fever, discharging sinuses, leukocytosis with left shift. Aspiration of the affected sites yielded predominantly pathogenic organisms at various sites.

Septic arthritis like osteomyelitis may result from haematogenous spread of bacteria or direct spread from a contiguous focus of osteomyelitis. Severe pain, tenderness, joint swelling, local warmth and marked limitation of motion are characteristic findings. However, septic arthritis must be differentiated from other arthropathy including synovial infection, synovitis associated with adjacent necrosis and nonspecific synovitis, which is usually self-limited and rarely progresses to chondrolysis.^13^

The history of patient, who presented with abdominal crisis due to microscopic infarction of bowel wall as a consequence of sickling within intestinal blood vessel, favors the speculation that anaerobic organisms escape from bowels into blood stream spreading into infarcted bones.^14^

This index SCD patient, who had refractory osteomyelitis displayed many complications like adhesive perivasculitis of the shoulder, avascular necrosis of the femoral and humeral head (refer fig 1&2) which explained why she was unable to walk and hence discharge on a wheelchair.

The commencement of hydroxyurea at the 20^th^ week of admission probably contributed to the speedy recovery of this patient. Hydroxyurea increases HbF level, reduces leukocytosis and thrombocytosis together with their attendant inflammatory and endothelia adhesive reactions and thereby curtail the inflammatory cascade that progresses to the generation of free radicals and to antioxidant consumption^15^

In conclusion this case has shown that life-threatening infections are major causes of morbidity and mortality in SCD, and the spectrum of organisms involved is largely determined by the source of infection, together with the kind and extent of existing immune abnormalities. The need for multidisciplinary approach is exemplified in this index case and this could be further actualized through establishment of well-equipped sickle cell Centers in Nigeria, which has the largest black population and the heaviest SCD burden in the world. An integrated rehabilitation center would assist patients like this to have improved functional capacity. Medical care of SCD requires heavy subsidy to allow optimized care. Infection control(immunization inclusive), early detection through high index of suspicion and adequate treatment using very potent antibiotics will to a great extent limit all these infections and their attendant complications.
